# Anti-Aging Effect of Urolithin A on Bovine Oocytes *In Vitro*

**DOI:** 10.3390/ani11072048

**Published:** 2021-07-09

**Authors:** Élisa Fonseca, Carla Cruz Marques, Jorge Pimenta, Joana Jorge, Maria Conceição Baptista, Ana Cristina Gonçalves, Rosa M. L. N. Pereira

**Affiliations:** 1 Instituto Nacional de Investigação Agrária e Veterinária, Quinta da Fonte Boa, 2005-048 Santarem, Portugal; elisa.fonseca1802@gmail.com (É.F.); carla.marques@iniav.pt (C.C.M.); jorge.pimenta@iniav.pt (J.P.); conceicao.baptista@iniav.pt (M.C.B.); 2 Centro de Investigação Interdisciplinar em Sanidade Animal, Universidade de Lisboa, Av. da Universidade Técnica, 1300-477 Lisboa, Portugal; 3 Group of Environment, Genetics and Oncobiology, Faculty of Medicine, Coimbra Institute for Clinical and Biomedical Research, University Coimbra, 3000-548 Coimbra, Portugal; jjorge@fmed.uc.pt (J.J.); acgoncalves@fmed.uc.pt (A.C.G.); 4 Center for Innovative Biomedicine and Biotechnology, University Coimbra, 3004-504 Coimbra, Portugal

**Keywords:** oocyte, aging, Urolithin A, assisted reproductive technologies

## Abstract

**Simple Summary:**

Post-ovulatory and maternal oocyte aging impair female reproductive capacity through several mechanisms that are not fully understood. Urolithin A (UA) is a natural compound previously identified to exert an anti-aging effects in several cells, which has never been used in bovine germinal cells. Our goal was to study UA effect on the developmental potential of the female gamete and the surround cumulus cells obtained from young and adult cows. A model for *in vitro* aging of female gametes was implemented to study different problems associated with reproductive aging and fertility impairment. Results confirmed that aging exerts a harmful effect on oocyte quality measured by using different parameters and gene expression levels of cumulus cells. Moreover, UA supplementation was an effective way to prevent oocyte aging, improving the subsequent bovine embryonic development.

**Abstract:**

Oxidative stress and mitochondrial dysfunction have been associated with the age-related decline of oocyte quality and strategies for their prevention are currently quested. Urolithin A (UA) is a natural metabolite with pro-apoptotic and antioxidant effects, capable of preventing the accumulation of dysfunctional mitochondria in different aged cells. UA has never been tested in bovine oocytes. Our aim was to study the effect of UA on the developmental potential of cumulus-oocyte-complexes (COCs) and granulosa cells’ (GCs) expression of important genes related to reproductive competence. Nuclear maturation progression, mitochondrial membrane potential (MMP) and developmental competence of physiologically mature (22 h) and *in vitro* aged oocytes (30 h of IVM) obtained from prepubertal and adult females, either supplemented with UA or not were assessed. Additionally, the amount of mRNA of several genes *(NFE2L2*, *NQO1*, and *mt-DN5*) and the number of *mt-ND5* DNA copies were quantified in cultured GCs from prepubertal and adult females, either supplemented with UA or not. Our study confirmed the harmful effect of oocyte aging on the nuclear maturation progression, MMP, developmental competence and gene expression levels. UA treatment during *in vitro* maturation enhanced (*p* < 0.05) the maturation rate and subsequent developmental capacity of aged oocytes. A positive effect (*p* < 0.05) of UA on physiological maturation, MMP and embryonic development was also identified. UA also interfered on the expression profile of *NFE2L2* and *NQO1* genes in GCs cultures. Our findings demonstrate that UA supplementation is an effective way to prevent oocyte aging and improves the subsequent bovine embryonic development.

## 1. Introduction

Decline of female reproductive ability is one of the first physiological functions adversely affected by aging and thus considered as an emerging health problem worldwide [1,2]. In addition to several pathological problems, the age-associated decrease in female fertility is largely attributed to a decline in the ovarian reserve of oocytes [1,3,4,5] allied to a time-dependent deterioration of their quality [2]. This deterioration process can occur due to the exposure of oocytes to an aged ovarian microenvironment before ovulation. In addition, the female gamete is often subjected to post-ovulatory aging when the fertilization process does not occur within the best optimal span period, and the unfertilized oocyte remains in the oviduct or *in vitro* prior to insemination for extended periods [6]. The impairment of oocyte quality is a critical factor associated to the failure of assisted reproductive technologies (ART), since its quality is the main determinant for the embryo’s developmental potential after fertilization [7,8]. Ovulation asynchrony and aged oocytes were often reported to impair the success of artificial insemination and embryo production programs in the mare, cattle and sheep implying important economic losses [1,9,10]. Therefore, it is of primordial importance to study the mechanisms underlying oocyte aging, in order to design better therapeutic approaches to rescue fertility in several species, including humans, and also as a tool for genetic improvement in livestock. Particular attention must be devoted to improving the developmental capacity of oocytes from prepubertal cattle, which are often used to accelerate genetic gain and shorten generation intervals.

One of the major causes of impaired developmental competence in aged oocytes is the increase in oxidative stress, which induces mitochondrial dysfunction, DNA damage and spindle formation errors, influencing the oocyte quality [11]. It is well established that increased production of free radicals is a cause of cellular aging in several chronic diseases and also in reproductive biology, resulting in poor fertility outcomes [12]. The ovarian microenvironment, which includes oocytes and granulosa cells (GCs), provides an antioxidant defense mechanism able to regulate oxidative conditions and to maintain the oxidant/antioxidant balance [13]. However, during the aging process, the efficiency of antioxidant defenses to neutralize reactive oxygen species (ROS) is attenuated, thus increasing the level of oxidative stress. The Nuclear factor-E2-related factor 2 (Nrf2 or NFE2L2), also known as Nrf2/Kelch-like ECH-associated protein 1 (Keap1) pathway, is a dominant response cascade activated by oxidative stress [14]. This pathway is a cellular defense mechanism that cells have developed to cope with deleterious effects of oxidative stress. Under normal conditions, Nrf2 is negatively regulated by Keap1, held in the cytoplasm and maintained at low levels. When exposed to oxidants, Nrf2 is dissociated from Keap1, allowing its translocation in the nucleus where it binds to specific DNA sequences. These sequences, named antioxidant response elements (ARE), lead to the transcriptional activation of cytoprotective genes, such as NAD(P)H:quinone-oxidoreductase-1 (*NQO1*), heme oxygenase-1 (*HMOX1*), and glutamate-cysteine ligase catalytic subunit (*GCLC*) [15,16]. Previous studies showed that the activation of Nrf2-Keap1 signaling pathway decreases the oxidative stress damage by elevating antioxidant levels in human GCs and mouse ovaries [17,18]. However, its role in the female gamete aging process remains elusive, although it is clearly established that mitochondria are the main production site for ROS during this process [9,10].

Conversely, mitochondria are involved in several critical cellular functions and are fundamental for meeting the demand of energy production required during oocyte maturation and subsequent embryonic development [19]. Competent mitochondrial activity has been highly associated with higher contents of mitochondrial DNA (mt-DNA) and ATP generation [20], higher mitochondrial membrane potential [21] and maintenance of mitochondria quality and quantity through mitophagy [22]. Moreover, the mitochondrial activity has been suggested to be directly correlated with embryo viability and better fertility outcomes [23]. Increasing evidence supports that age-related mitochondrial alterations drive to ovarian aging and subsequently reduced embryo viability and implantation potential. These alterations include decreased mt-DNA copy number, decreased ATP generation [20], alterations in mitochondrial gene expression [24], mt-DNA damage [25] and reduced mitochondrial membrane potential [26].

Mitochondria-targeted therapeutic approaches prompted a huge interest for several pathologies associated with aging due to their great potential in enhancing mitochondrial function [27]. Within this framework, Urolithin A (UA)—a natural metabolite obtained after the ingestion of food such as pomegranates followed by its conversion by the gut microbiota—has been demonstrated to prevent the accumulation of dysfunctional mitochondria with age, inducing mitophagy and also maintaining mitochondrial biogenesis and respiratory capacity [28,29]. UA has been applied as a promising therapeutic drug to prevent some cancers, such as colorectal and prostate cancer [30,31]. Additionally, UA also has anti-inflammatory [32], anti-obesity [33], antioxidant [34] and anti-aging properties [35]. A recent study has highlighted the effects of UA supplementation to senescent human skin fibroblasts on the activation of Nrf2-Keap1 pathway enhancing their antioxidant capacity. This activation of the Nrf2-Keap1 pathway effectively mitigates the ROS level, through the upregulation of the expression of Nrf2 downstream ARE-response genes (*SOD, NQO1, GCLC* and *HMOX1*), indicating a promising anti-aging effect [35].

Several studies have been performed with the goal of delaying ovarian aging, consequently improving oocytes quality and the fertility outcomes. Due to the contribution of oxidative stress to the ovarian aging process, as well as to mitochondrial dysfunction, supplementation with antioxidants has appeared as a promising therapy [36,37]. However, it is unknown whether Urolithin A supplementation may restore the damage that occurs during ovarian aging contributing to prevent infertility problems. Therefore, the aim of this study was: (1) to demonstrate that aging could alter cumulus-oocyte-complexes’ (COCs) developmental potential and GCs’ expression of important genes involved in the Nrf2 signaling pathway; (2) to determine whether UA can rescue female fertility demonstrating an anti-aging effect in aged COCs and GCs; and (3) to evaluate UA effect on the expression level of genes involved in the Nrf2 signaling pathway as well as on oocyte quality.

## 2. Materials and Methods

### 2.1. Experimental Design

This study was approved by the Animal Care Committee of the National Veterinary Authority (N°08965DGAV), following European Union guidelines (no. 86/609/EEC). To investigate the effect of aging on the alteration of oocyte quality and the potential anti-aging effect of UA, a model using COCs collected from prepubertal and adult cows submitted to *in vitro* aging (30 h of maturation) or to the physiological maturation (22 h) processes were applied.

#### 2.1.1. Previous Assay—Dose-Response Study

A previous assay to determine the concentration of UA that should be used during the bovine COCs maturation process was performed based on a dose-response study in four sessions. Since UA has never been tested in bovine oocytes, previous doses successfully applied for prevention and mitigation of some cancers and to demonstrate the anti-aging effect of UA in different cell lines were used [28,38,39]. COCs obtained from prepubertal and mature adult cows (n = 978) were selected and then randomly divided into five groups to test different doses of UA: control, 1, 10, 25, and 50 μM during physiological *in vitro* maturation. After the maturation period, some oocytes (n = 154) were stained to determine the chromosomal configuration and maturation stages. The remaining matured oocytes were submitted to *in vitro* insemination with frozen/thawed semen. Presumptive zygotes were cultured, and cleavage and blastocyst rates were determined at day 2 and day 7 of culture, respectively. Based on the obtained results, namely the absence of harmful effects and the promotion of maturation and blastocyst development, the concentration of 1 μM of UA was selected. 

#### 2.1.2. Experiment 1

In this experiment, carried out in six sessions, both COCs from prepubertal (mean age = 9 months, n = 660) and adult (mean age = 39 months, n = 674) cows were collected to assess the oocyte quality and developmental potential of aged and physiologically matured oocytes as well as UA effect to rescue female fertility. COCs were randomly divided into 8 groups: (1) control prepubertal group, COCs from prepubertal calves matured for 22 h (n = 148); (2) UA prepubertal group, COCs from prepubertal calves matured in medium supplemented with 1 μM of UA for 22 h (n = 155); (3) control aged 30 h prepubertal group, COCs from prepubertal calves aged through 30 h of *in vitro* maturation (n = 149); (4) UA aged 30 h prepubertal group, COCs from prepubertal calves aged *in vitro* for 30 h in maturation medium supplemented with 1 μM of UA (n = 144); (5) control adult group, COCs from adult cows matured for 22 h (n = 155); (6) UA adult group, COCs from adult cows matured in medium supplemented with 1 μM of UA for 22 h (n = 129); (7) control aged 30 h adult group, COCs from adult cows aged through 30 h of *in vitro* maturation (n = 148); and (8) UA aged 30 h adult group, COCs from adult cows aged *in vitro* for 30 h in maturation medium supplemented with 1 μM of UA (n = 138). After the respective *in vitro* maturation periods, oocytes were inseminated with thawed capacitated bull semen. Subsequently, embryonic development was assessed, evaluating both the rate of cleaved and produced embryos, as well as their quality.

Additionally, in this experiment, COCs from each group were retrieved to assess their nuclear maturation stage (control prepubertal group, n = 7; UA prepubertal group, n = 7; control aged 30 h prepubertal group, n = 10; UA aged 30 h prepubertal group, n = 6; control adult group, n = 16; UA adult group, n = 21; control aged 30 h adult group, n = 16; UA aged 30 h adult group, n = 18). The mitochondrial membrane potential (MMP) of COCs was also evaluated (control prepubertal group, n = 10; UA prepubertal group, n = 9; control aged 30 h prepubertal group, n = 9; UA aged 30 h prepubertal group, n = 9; control adult group, n = 15; UA adult group, n = 13; control aged 30 h adult group, n = 10; UA aged 30 h adult group, n = 14). 

#### 2.1.3. Experiment 2

As the GCs play an essential role in follicular growth and oocyte development, a second experiment was performed in five sessions to further study the effect of age and UA on the expression of *NFE2L2*, *NQO1* and *mt-ND5*. The number of copies of *mt-ND5* gene was also evaluated. GCs were obtained after centrifugation of the follicular fluid aspirated from ovaries of prepubertal (mean age = 10 months) and adult cows (mean age = 62 months). These cells were cultured in the following conditions: (1) prebubertal control, culture of GCs of prepubertal calves; (2) prepubertal UA, culture of GCs of prepubertal calves supplemented with 1 μM UA; (3) adult control, culture of GCs of adult cows; and (4) adult UA, culture of GCs of adult cows supplemented with 1 μM UA. After GCs’ confluence at the 5th day of culture, they were snap frozen in liquid nitrogen and later the DNA and RNA were extracted, allowing the subsequent quantification of *NFE2L2*, *NQO1* and *mt-ND5* mRNA transcripts and also *mt-ND5* copies number.

### 2.2. Oocyte Collection and In Vitro Maturation

Ovaries from adult and prepubertal cows (previous assay, n = 978 and exp. 1, n = 1334) were collected at a local slaughterhouse, and kept at 35–37 °C, in a phosphate-buffered saline (PBS) supplemented with 0.15% of bovine serum albumin (*w*/*v*, BSA) supplemented with 0.05 mg mL^−1^ of kanamycin. At the laboratory, ovarian follicles with 2–8 mm in diameter were aspirated with a 19-gauge needle. Only COCs with at least three layers of compact cumulus cells and a homogeneous ooplasm were washed and selected for maturation according to the experimental design. Maturation was accomplished in an incubator at 38.8 °C, 5% CO_2_ in humidified air for 22 or 30 h in a maturation medium composed of tissue culture medium 199 (TCM) with 10% of fetal bovine serum, 0.2 mM sodium pyruvate, 10 ng mL^−1^ of epidermal growth factor, and 10 μL mL^−1^ of gentamicin [40].

### 2.3. Granulosa Cells Collection and Culture

Granulosa cells were obtained from the recovered follicular fluid after centrifugation for 10 min at 200× *g* [41]. The pellet was suspended in 1 mL of culture medium (TCM199 + 10% serum) to perform another centrifugation for 5 min. The new pellet was resuspended in 1 mL of culture medium either supplemented with 1 μM of UA or not according to the experimental design and homogenized with a syringe attached to a 19G-needle, at least 30 times to detach the cells. After evaluation of GC viability (tripan blue dye, 0.4% *w*/*v*), cells were seeded at a concentration of 2 × 10^5^ viable cells mL^−1^ and cultured for five days at 38.8 °C, 5% CO_2_ in a humidified atmosphere until confluence. At every 48 h, the culture medium was discharged and refreshed with a new one. For DNA and RNA extraction, GCs were collected and washed by centrifugation at 200× *g* for 10 min. Cell pellets were resuspended in 1 mL of PBS, immediately snap frozen in liquid nitrogen, and stored at −80 °C.

### 2.4. Oocyte Nuclear Maturation

Nuclear maturation stages were assessed following the 22 h or 30 h period of *in vitro* maturation. Denuded oocytes were fixed in an acetic acid/ethanol (1:3, *v*/*v*) solution, and maintained at 4 °C for 48 h. Then oocytes were stained with 1% aceto-lacmoid solution, mounted in a Neubauer chamber and observed under a phase contrast microscope (Olympus BX41). Oocytes were classified as follows: Germinal Vesicle (GV), Condensing Chromosomes I (CCI), Condensing Chromosomes II (CCII), Diakinesis, Anaphase-I/Telophase-I (AI/TI), and MII (Metaphase-II). Only oocytes with visible chromatin staining were taken into account [42].

### 2.5. Assessment of Mitochondrial Membrane Potential

To measure the mitochondrial membrane potential (MMP), an indicator of mitochondrial activity, mitochondria were stained with 5, 5′, 6, 6′-tetrachloro-1, 1′, 3, 3′-tetraethylbenzimidazolcarbocyanine iodide (JC-1, Invitrogen, Waltham, MA, USA). Denuded oocytes were incubated with 5 μg mL^−1^ of JC-1 [37] in maturation medium for 30 min at 38.8 °C and 5% CO_2_ in humidified air in the dark. Oocytes were washed twice in PBS and immediately transferred to a pre-heated slide glass and observed under a fluorescence microscope (Olympus BX51) using the blue fluorescence filter (BP 470–490, objective UPlanFI 20×/0.50). Mitochondrial membrane potential was then calculated as the ratio of the measured red/green fluorescence using the ImageJ software (National Institute of Health, Bethesda, MD, USA).

### 2.6. In Vitro Fertilization and Embryo Culture

*In vitro* fertilization was performed with frozen-thawed sperm of a Holstein-Frisian bull, previously submitted to capacitation using the Percoll gradient (45 and 90) method, at a concentration of 2 × 10^6^ spermatozoa mL^−1^. COCs and sperm were co-incubated for 20 h at 38.8 °C and 5% CO_2_ in humidified air. Presumptive zygotes were then transferred to droplets of synthetic oviductal fluid (SOF) medium supplemented with BME and MEM amino acids, glutamine, glutathione, and BSA [40]. After 48 h of the insemination, the cleavage rate (cleaved embryos per total inseminated oocytes) was assessed, and cleaved embryos were maintained in SOF supplemented with BSA and 10% of fetal bovine serum (FBS). Embryos were cultured for 12 days [41,43] to assess the blastocyst development rate (at days 7, 9, and 12; D7 embryos per cleaved embryos) and hatched embryo rate (hatched embryos per D7 embryos) and their quality [43]. Day 7 embryos were classified as grade 1 (good quality), 2 (fair quality), and grade 3 (bad quality) [40,44].

### 2.7. DNA and RNA Extraction and Quantification

Total DNA and RNA were isolated from GCs using the High Pure PCR Template Preparation Kit (Roche, Basel, Switzerland) and PureLink™ RNA Mini Kit (Invitrogen™, Waltham, MA, USA), respectively, according to the manufacturer’s instructions. Those protocols included the use of spin columns used to isolate high-quality total DNA and RNA, and DNase as treatment to remove genomic DNA from RNA [40]. After extraction, the samples were stored at −80 °C. The concentration and quality of DNA and RNA were determined using a NanoDrop™ One/OneC Spectrophotometer (ThermoFisher Scientific™, Waltham, MA, USA).

#### 2.7.1. Complementary DNA Synthesis

Synthesis of complementary DNA (cDNA) from RNA isolates were performed using the Xpert cDNA Synthesis Mastermix kit (GRiSP, Porto, Portugal, according to the manufacturer’s instructions. RNA was reverse transcribed using 500 ng of extracted RNA from each sample to perform cDNA synthesis, which was carried out using a thermocycler (T100 Thermal Cycler, Bio-Rad, Hercules, CA, USA). The resultant cDNAs were stored at −20 °C until use for further assays.

#### 2.7.2. Primer Design

For this study, primers for the targeted (*NFE2L2* and *NQO1*) and an endogenous control gene (*β-actin*) were designed using the Primer BLAST software of the National Center for Biotechnology Information (NCBI) (http://www.ncbi.nlm.nih.gov/tools/primer-blast/, accessed on 6 March 2020). Sequences of primers for the reference genes and the target genes are depicted in Table 1. Additionally, the *mt-ND5* gene was used in this work and details about *mt-ND5* primers were retrieved from a previous study [45].

#### 2.7.3. Quantitative Reverse-Transcription Polymerase Chain Reaction

Real-time PCR analyses were performed using the Xpert Fast SYBR Green Mastermix 2X with ROX in a QuantStudio 3 thermocycler (ThermoFisher Scientific™, Waltham, MA, USA), using cDNA at a concentration of 25 ng uL^−1^. The assessment of the mitochondrial DNA (mt-DNA) copies number of the *ND5* gene was carried out by qPCR through the previously extracted DNA, using the same equipment as for the quantification of genes. Each optimized reaction was performed, consisting of Xpert Fast SYBR Green Mastermix 2X with ROX, primer (Forward and Reverse) of each target gene, sample (cDNA/DNA) and RNase free water making up a total volume of 10 μL. The samples were analyzed in duplicate and reactions containing water instead of template were included as negative controls. The samples were subjected to an amplification protocol that consisted of an initial cycle at 95 °C for 2 min of denaturation phase, followed by 40 denaturation cycles at 95 °C for 5 s, 40 annealing cycles for 30 s at 60 °C (depending on the melting temperature of primer sequences), and extension phase at 72 °C for 30 s and, lastly, a final extension period at 72 °C for 10 min. 

For the gene expression quantification, the relative quantification method was used. This method of relative quantification of gene expression was carried out with the expression levels of the target genes under study, which were normalized with the housekeeping genes, by the CT comparative method. As the amplification by RT-qPCR was performed in duplicate, the mean CT values for each gene were determined and the expression levels were calculated using the following formula:

2(^−ΔCt^), (1)

where ΔCt = Ct target gene − Ct endogenous control gene.

For the quantification of the mt-DNA number of copies, the relative quantification normalized against unit mass method was used. This method was carried out with the CT values of GCs treated with UA (named as test), which were normalized with control samples (currently designated as calibrator). As the *mt-ND5* copy number was assessed by qPCR and performed in duplicate, the mean CT values for the tests and calibrators samples were determined and the ratios were calculated using the following formula:

Ratio = E(^ΔCt^), (2)

where ΔCt = Ct calibrator − Ct test, and E is the efficiency.

### 2.8. Statistical Analysis

Data from embryo production and quality sessions were analyzed by using Proc Glimmix from SAS (Statistical Analysis Systems, SAS Inst., Inc., Cary, NC, USA), using the binary distribution and the logit as link function. The generalized linear mixed model included treatment (UA doses, aging effect and female age, and their interaction) as fixed effect and replicates as random effect. In addition, the means for each treatment were calculated, and comparisons between groups were performed using the PDIFF test. The JC-1 data, mRNA transcript levels and mt-DNA number of copies were analyzed using the Proc Mixed of SAS with a model including treatment (UA effect, aging effect, and/or female age, and their interaction) as fixed effect. The session was considered a random effect. The data of chromosomal configurations were compared between groups using the exact Fisher test, in a 2 × 2 contingency table. The analysis of results was considered statistically significant when *p* < 0.05.

## 3. Results

### 3.1. Previous Assay—Dose-Response Study

**Figure 1:**
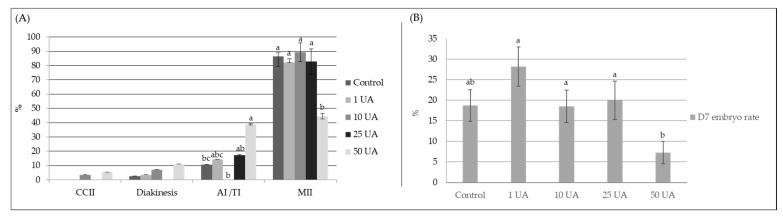
Effect of Urolithin A (UA) doses (control, 1 UA, 10 UA, 25 UA, and 50 UA, correspond to 0, 1, 10, 25 and 50 μM of UA, respectively) supplemented to the maturation medium on: (**A**) oocyte chromosomal configuration. Different letters indicate significant differences between groups for the same stage (CCII, condensing chromosomes II; AI/TI, Anaphase-I/Telophase-I; MII, Metaphase-II, *p* ≤ 0.05); (**B**) D7 embryo rate. Different letters indicate significant differences between groups (*p* ≤ 0.05). D7, day 7.

The oocyte maturation status observed during the dose-response study of UA supplemented to the maturation medium is represented in Figure 1. This supplementation significantly influenced the nuclear maturation stages of oocytes. The 50 UA group (38.9 ± 0.5%) revealed a higher rate of oocytes in the AI/TI phase status compared to the Control (10.8 ± 0.5%, P = 0.03) and 10 UA (0.0 ± 0.0%, *p* = 0.0006) groups. In this phase, a higher rate in the 25 UA (17.2 ± 0.5%, *p* = 0.05) group compared to the 10 UA group was also identified. Moreover, a trend was found between 50 UA group and 1 UA (14.3 ± 2.0%, *p* = 0.08). The highest dose also showed a harmful effect, reducing the number of oocytes classified at the Metaphase-II (MII) stage (50 UA, 44.4 ± 2.0%) when compared to control (86.5 ± 7.0%, *p* = 0.003), 1 UA (82.1 ± 2.5%, *p* = 0.01), 10 UA (89.3 ± 6.5%, *p* = 0.002) and 25 UA (82.8 ± 9.0%, *p* = 0.01) groups. No differences were observed between the remaining stages of oocyte maturation. The GV and CCI maturation phases were not shown in the Figure 1, as no oocytes were assessed in these categories.

The dose-response test showed no significant effect of UA concentrations on cleavage and hatched embryo rates. However, the rate of embryos produced at day 7 was influenced by the different concentrations of UA. The 50 UA group (7.2 ± 2.7%) had lower rates of embryos at day 7 compared to the 1 UA (28.5 ± 4.8%, *p* = 0.004), 10 UA (20.4 ± 4.0%, *p* = 0.03) and 25 UA (18.5 ± 4.7%, *p* = 0.05) groups. Although no significant differences were found between the control and 50 UA groups, a trend (*p* = 0.06) was identified. Moreover, day 7 embryo rates tend (*p* = 0.07) to be higher after 1 UA dose supplementation compared to control. Moreover, the 1 UA group doubled the number of excellent/good embryo quality (68.9% of grade 1 embryos, n = 20) when compared to control (34.6%, n = 10) and 10 UA (26.8%, n = 10). A low number of grade 1 embryos was obtained with the higher doses: 25 UA group (15.4%, n = 4) and 50 UA (35.4%, n = 3). Based on the above-mentioned results, the 1 μM UA was chosen to proceed to the following studies.

### 3.2. Experiment 1—Oocyte Quality and Developmental Potential of Aged and Physiologically Matured Oocytes

#### 3.2.1. Nuclear Maturation

We investigated the effect of UA supplementation on chromosomal maturation status of aged and physiologically matured oocytes collected from prepubertal and adult females. Independently of the UA effect, oocyte aging and female age induced a significant harmful effect on maturation. Higher rates of MII phase were identified in control oocytes compared to aged ones (22 h = 93.2 ± 5.4% vs. 30 h = 77.6 ± 4.1%, *p* = 0.02). Moreover, a delay on maturation progression was identified on aged gametes and prepubertal females with more oocytes at AI/TI phase (22 h = 3.4 ± 0.5% vs. 30 h = 17.9 ± 0.6%, *p* = 0.01 and prepubertal = 21.1 ± 1.1% vs. adult = 6.8 ± 0.7%, *p* = 0.03). Moreover, a higher number of degenerated oocytes after 30 h of IVM was identified compared to after 22 h (7.1 ± 1.39% vs. 4.1 ± 0.89%, *p* = 0.02). The female age and UA supplementation did not interfere (*p* > 0.05) with the number of degenerated oocytes.

**Figure 2:**
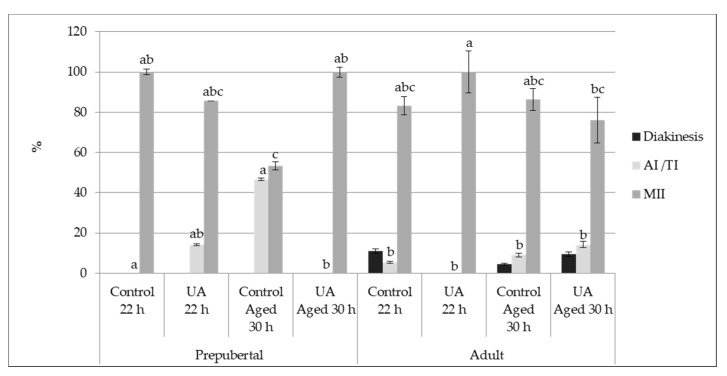
Effect of the Urolithin A (UA) supplementation to the maturation medium, cumulus-oocyte-complexes (COCs) aging and female age on the oocyte chromosomal configuration (prepubertal: control 22 h, UA 22 h, control aged 30 h, UA aged 30 h; adult: control 22 h, UA 22 h, control aged 30 h, UA aged 30 h groups). Different letters indicate significant differences between groups for the same stage (AI/TI, Anaphase-I/Telophase-I; MII, Metaphase-II) (*p* ≤ 0.05).

Both *in vitro* aging and UA supplementation to the maturation medium significantly influenced the chromosomal configuration of oocytes from prepubertal and adult females (*p* < 0.05, Figure 2). On the AI/TI phase of the maturation status, higher rates of oocytes were found in this stage in the control aged 30 h prepubertal group (46.7 ± 0.5%) when compared to control prepubertal (0.0 ± 0.0%, *p* = 0.05) and UA aged 30 h prepubertal (0.0 ± 0.0%, *p* = 0.02), and both adult control (5.6 ± 0.5%, *p* = 0.01 and UA adult, 0.0 ± 0.0%, *p* = 0.0002) and control aged 30 h adult (9.1 ± 1.0%, *p* = 0.02) groups. A trend was also identified between UA adult and UA aged 30 h adult (*p* = 0.0769).

Regarding the MII phase corresponding to a complete nuclear maturation, significant differences were also found between groups (Figure 2). Lower rates of oocytes on the MII stage were found in the control aged 30 h prepubertal (53.3 ± 2.0%) compared to the control prepubertal (100.0 ± 1.5%, *p* = 0.05), UA aged 30 h prepubertal (100.0 ± 2.5%, *p* = 0.02) and UA adult (100.0 ± 10.5%, *p* = 0.0002) and control aged 30 h adult (86.0 ± 5.5%, *p* = 0.056) groups. Moreover, the UA adult group showed higher rates of matured oocytes (MII) compared to the UA aged 30 h adult (76.2 ± 11.3%, *p* = 0.01), control aged 30 h adult (86.0 ± 5.5%, *p* = 0.08) and control adult (83.3 ± 4.5%, *p* = 0.057).

No differences were observed between the remaining stages of oocyte maturation.

In summary, UA supplementation improved *in vitro* maturation progression in oocytes submitted to the aging process, especially in prepubertal females. An important anti-aging effect of UA was thus identified.

#### 3.2.2. Mitochondrial Membrane Potential

In order to analyze the involvement of mitochondrial dysfunction in female aged oocytes and the effect of UA and maternal age, the mitochondrial membrane potential (MMP) was assessed in oocytes of different groups.

Independently of the female age and UA effects, oocyte aging had a significant harmful effect on MMP assessed as the ratio of the measured red and green fluorescence (22 h ratio = 0.54 ± 1.8% vs. 30 h ratio = 0.47 ± 1.5%, *p* = 0.002).

**Figure 3:**
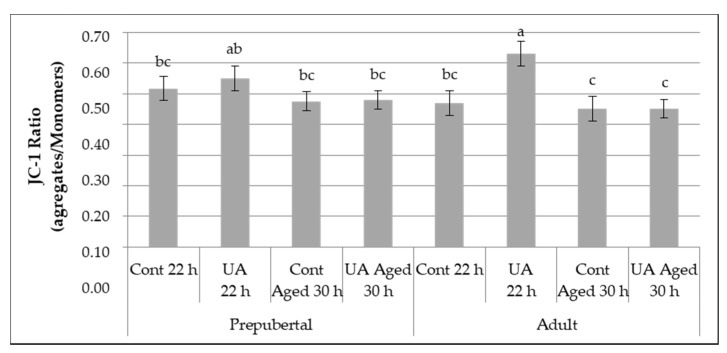
Effect of the Urolithin A (UA) supplementation to the maturation medium, cumulus-oocyte-complexes (COCs) aging and female age on mitochondrial membrane potential (MMP). The assessment of mitochondrial activity was performed using the average of the ratios (aggregate/monomers) for each oocyte analyzed in each group (prepubertal: control 22 h, UA 22 h, control aged 30 h, UA aged 30 h; adult: control 22 h, UA 22 h, control aged 30 h, UA aged 30 h groups). Different letters indicate significant differences between groups (*p* < 0.05). Data are expressed as the mean ratios ± standard error mean (SEM).

The combination of the female age, aging effect and the supplementation with UA during maturation significantly (*p* = 0.007) influenced the MMP (Figure 3). As observed in Figure 3, higher rates of MMP were obtained in the UA prepubertal (ratio = 0.55 ± 0.04) and UA adult (ratio = 0.63 ± 0.04) groups where oocytes were matured for 22 h, compared with the control aged 30 h adult (ratio = 0.45 ± 0.04, *p* = 0.04 and *p* = 0.0007, respectively) and UA aged 30 h adult (ratio = 0.45 ± 0.03, *p* = 0.03 and *p* = 0.0003, respectively) groups. Moreover, a significant increase in JC-1 aggregate/monomers ratio, which indicates a significant increase in MMP, was observed in the UA adult group when compared with control adult (ratio = 0.47 ± 0.04, *p* = 0.0031), control prepubertal (ratio = 0.52 ± 0.04, *p* = 0.03), control aged 30 h adult (ratio = 0.48 ± 0.03, *p* = 0.003) and UA aged 30 h adult (ratio = 0.47 ± 0.03, *p* = 0.001) groups. A trend was also identified between UA prepubertal, and UA aged 30 h prepubertal (*p* = 0.07).

In summary, UA improved the MMP during the physiological maturation but was not capable of overcoming the negative effect of aging in both prepubertal and adult oocytes.

#### 3.2.3. Embryonic Development

The embryonic developmental potential was evaluated through cleavage, D7 and hatched embryo rates, obtained with the different treatments (aging and UA supplementation) applied to the prepubertal and adult females COCs during maturation. Independently of the female age and UA effects, oocyte aging had a significant harmful effect on cleavage (22 h = 79.5 ± 1.7% vs. 30 h = 68.6 ± 2.0%, *p* = 0.0001) and D7 embryo rates (22 h = 18.6 ± 1.9% vs. 30 h = 12.1 ± 1.7%, *p* = 0.01). The UA supplementation was also shown to improve the D7 embryo rates (control = 11.1 ± 1.5% vs. UA = 20.0 ± 2.0%, *p* = 0.0009).

The combination of the female age, aging effect and the supplementation with UA during maturation significantly (*p* = 0.01) influenced the embryonic development, namely the cleavage and D7 embryo rates (Table 2). Higher rates of cleavage were achieved when the adult oocytes were matured for 22 h with or without UA supplementation (control adult = 1.4 ± 3.2% and UA adult = 80.9 ± 3.4%) and prepubertal oocytes matured for 22 h with UA supplementation (UA prepubertal = 80.6 ± 3.2%), compared to UA aged 30 h adult (66.5 ± 4.2%, *p* ≤ 0.01), control aged 30 h prepubertal (66.9 ± 3.9%, *p* ≤ 0.02) and UA aged 30 h prepubertal (67.2 ± 4.0%, *p* ≤ 0.01) groups (Table 2). A trend (*p* = 0.09) was also identified between the control adult and the control aged 30 h adult groups.

Another parameter that was significantly (*p* = 0.01) influenced by the combination of the female age, the supplementation with UA and COCs aging was the rate of embryos produced at day 7 (Table 2). Exception made for the UA prepubertal and UA 30 h prepubertal groups, the UA adult group presented the highest rates of embryo at day 7 (26.8 ± 4.3%, *p* ≤ 0.05). Moreover, lower D7 embryo rates were produced from aged oocytes from both adult and prepubertal females without the supplementation of UA (control aged 30 h prepubertal = 7.7 ± 2.6% and control aged 30 h adult = 7.7 ± 2.6%) compared to those that were supplemented with UA, respectively (UA aged 30 h prepubertal = 18.9 ± 4.0%, *p* = 0.03 and UA aged 30 h adult = 15.9 ± 4.0%, *p* = 0.09). The supplementation of UA to the maturation medium did not significantly (*p* > 0.05) influence the quality rates of produced embryos of grade 1, 2 and 3, nor did the aging effect or all the studied effects together (Table 3). However, the number of embryos of excellent/good quality that were produced when the UA were supplemented to oocytes matured for 22 h doubled (Table 3).

### 3.3. Experiment 2

#### 3.3.1. Gene Expression Levels

In order to study the UA effect and maternal age influence in the Nrf2 signaling pathway, analysis of *NFE2L2* and *NQO1* genes expression levels in GCs culture from prepubertal and adult cows was assessed by RT-qPCR. Independently of the other studied factors, female age significantly influenced the *NFE2L2* gene expression, reflected in the higher level of *NFE2L2* transcripts in GCs from prepubertal females (mRNA levels prepubertal = 0.00015 ± 0.0012% vs. adult = 0.00011 ± 0.0012, *p* = 0.048).

**Figure 4:**
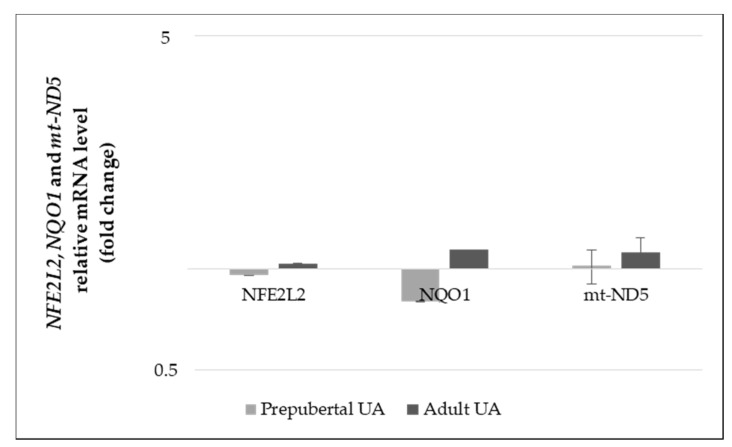
Gene expression levels of *NFE2L2*, *NQO1* and *mt-ND5* in granulosa cells (GCs) from prepubertal and adult cows (prepubertal: control, Urolithin A (UA); adult: control, UA, n = 5 for each group) supplemented with UA in the medium culture. Results are normalized to the β-actin gene. mRNA levels in control GCs for *NFE2L2, NQO1* and *mt-ND5* genes were set to 1. Data are expressed as the CT value mean ± standard error mean (SEM).

The supplementation of UA to the culture medium of GCs, both independently and considering female age, did not significantly (*p* > 0.05) influence the gene expression levels of *NFE2L2*, *NQO1* and *mt-ND5* genes. Figure 4 represents the expression levels of these genes that were normalized with the respective control, in both UA treated prepubertal and adult GCs.

#### 3.3.2. mt-DNA Copy Number

**Figure 5:**
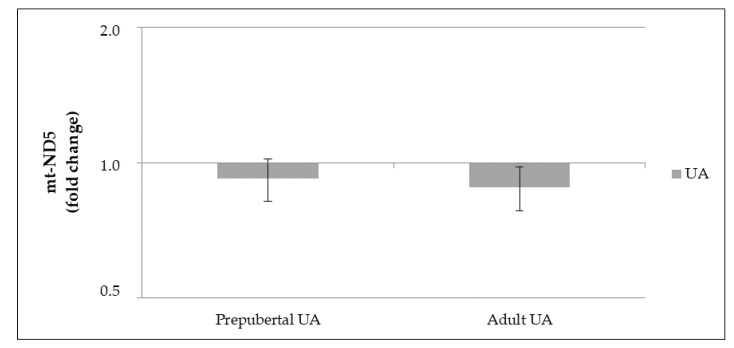
mt-ND5 copy number in granulosa cells (GCs) from prepubertal and adult cows (prepubertal: control, Urolithin A (UA); adult: control, UA, n = 5 for each group) supplemented with UA in the medium culture, assessed by qPCR using DNA. Results are normalized to their respective GCs control samples. mt-DNA copy numbers in control GCs for ND5 gene were set to 1. Data are expressed as the ratio of CT values mean ± standard error mean (SEM).

Analysis of mt-ND5 DNA content in GC culture from prepubertal and adult cows were assessed by qPCR. The supplementation of UA to the culture medium of GCs, both independently and considering female age, did not significantly (*p* > 0.05) influence the copy number of mt-ND5 gene. Figure 5 represents the copy number of this gene normalized with the respective controls, in both UA treated prepubertal and adult GCs.

## 4. Discussion

Over the past decades, an increasing demand for ART application in livestock has been observed [8,46,47]. Additionally, delayed childbearing associated to advanced maternal age has now become the main factor leading women to resort to ART [5]. Oocyte quality is critical for the occurrence of a successful conception, and aging of the female gamete a major concern for ART success. Despite the increasing advances made in this field, the applied technologies are not yet able to restore fertility, reverting the biological clock [7]. In the present work, a model for the study of age-associated infertility in cattle oocytes was developed. Due to the similarities to human pregnancy, follicular and endocrine events [48,49], this model may also be useful for human research, avoiding the ethical and physical restrictions that hamper these studies in human oocytes. Our model for aging female gametes proved to be very efficient and suitable for studying different problems associated with reproductive aging and fertility impairment, currently one of most critical challenges in the world. Furthermore, the search for new upcoming antioxidant therapies with the potential to prevent infertility provoked by the female gamete aging, as studied in the present work, is equally of the utmost importance.

Presented results reported for the first time the beneficial effect of UA in ART outcomes. UA is a food metabolite capable of preventing the accumulation of age-related dysfunctional mitochondria by inducing their mitophagy and an extended lifespan of cells [28]. Since UA has never been tested before in bovine reproduction, doses successfully applied in different cell lines showing its anti-cancer, anti-inflammatory and anti-aging effects were used [28,35,39]. In our study, a previous dose-response assay was carried out and the concentration of 1 µM UA was clearly identified as the most promising. Moreover, a deleterious effect was demonstrated at higher doses, especially at the 50 µM UA dose, harming the progression of nuclear maturation until MII. This effect was reflected on embryonic development impairment. Accordingly, Liu and colleagues (2019) noticed significant reduced cell viability and proliferation of human senescent skin fibroblasts, at a UA concentration of 50 µM. They referred that high UA doses lead to diminished cell viability increasing the number of cells arrested in the G/M cell-cycle [35]. On the contrary, higher rates of embryos were produced at day 7 after the supplementation of 1 µM UA to the maturation medium when compared to the other doses. To further explore the potential mechanisms of the action of UA in female reproduction, a study during oocyte aging and using females with different ages as oocyte donors was implemented. Yamamoto and co-workers (2010) reported an age-associated decline in the fertilization rate of old cows, showing that these oocytes were more prone to resume first meiotic division during maturation and often had already initiated the meiotic maturation at oocyte collection [50]. These data suggested that oocytes from older cows had a faster nuclear maturation progression and reached the MII phase faster than oocytes from young females due to a lower oocyte competence [51]. Accordingly, our study reveals that female donors’ age and the process of oocyte aging significantly influence the chromosomal configuration of oocytes during maturation progress. In fact, oocytes from prepubertal cows showed a higher rate of delayed stages, such as AI/TI phases (prepubertal = 21.1% vs. adult = 6.8%), revealing a slower progression of nuclear maturation, which may be due to lower oocyte competence as proposed by Soto-Heras and colleagues (2018). Aged oocytes also present lower rates of oocytes that have reached the MII phase during a 30 h period (77.6%) compared to the physiological period (93.2%). Moreover, a positive effect has been identified with UA treatment during the physiological maturation process, and also an anti-aging effect in both prepubertal and adult females.

In agreement with our findings, a study has demonstrated that melatonin supplementation during the *in vitro* maturation could stimulate the meiosis resumption in bovine COCs, whereas control oocytes cultured without hormones had slower meiosis resumption rates [52]. On the contrary, the supplementation with other antioxidant agents, such as quercetin, vitamin C or resveratrol did not present any effect on nuclear maturation rates, even when a reduced ROS levels or increased antioxidant enzymatic levels were observed [53,54]. Conversely, our results show that UA could rescue oocytes from aging effects in both aged adult and prepubertal females, which present lower competence, improving maturation rates.

One of the main contributors to poor fertility outcomes affecting oocyte quality is related to mitochondrial functions, which become compromised with advanced maternal age and post-ovulatory aging [21,55]. The MMP have been widely studied in different models, revealing that both aged gametes after ovulation and maternal aging, induce the loss of mitochondrial function [56]. Consequently, the loss of MMP was negatively reflected on oocyte and embryo development [26,57]. According to these findings, in our study, a reduced MMP ratio was found in aged oocytes from both prepubertal and adult cows. Moreover, UA supplementation to the culture medium induced an increase on MMP of prepubertal and adult oocytes matured for 22 h, reverberating in higher cleavage rates. These data are in agreement with the results found by Liang and co-workers (2017) which observed a MMP enhance when bovine oocytes were supplemented with melatonin for 22 h [26]. However, we also identified a reduction in MMP in aged oocytes supplemented with UA, denoting that UA may not be able to reverse the negative effect of aging in MMP. Discrepant results have been observed concerning the MMP after supplementation of aged oocytes with antioxidant compounds. Indeed, several authors reported greater levels of MMP on oocytes after supplementation with melatonin [26] and laminarin [21], whereas others have observed the opposite, a decreased MMP in aged oocytes treated with L-carnitine [58] and melatonin [38]. Additionally, Ryu and colleagues (2016) showed a reduction in MMP in mice myoblasts cultured with UA [28]. Regarding our results, further studies should be addressed to deepen the knowledge of the mechanism of action of UA in the oocyte mitochondria and explain the identified differential effects on aged and physiologically matured oocytes.

Intrinsic quality of oocytes has been widely reported as the main determinant of subsequent embryonic development. Accumulated evidence revealed that several cellular and molecular abnormalities occur during extended *in vitro* maturation periods as well as during *in vivo* post-ovulatory aging [57]. Furthermore, these abnormalities can exert relevant impacts on oocyte quality reverberating on embryo production [52]. To demonstrate that UA can act as an anti-aging compound and improve oocyte quality, delaying oocyte aging, we investigated the developmental capacity of aged oocytes after *in vitro* fertilization. In our study, a significant (*p* ≤ 0.01) harmful effect of gamete aging on cleavage and day 7 embryo production rates were observed. Accordingly, several authors referred that old females have an age-associated decline in reproductive capacities, reflected in lower fertility rates and poor embryo quality [44,55]. On the other hand, previous studies testing other antioxidant molecules to rescue aged oocytes have reported the beneficial effect of a few of these compounds during *in vitro* maturation on the embryo development of aged females from different species, such as cattle [58], pig [38] and mice [6]. For instance, L-carnitine was tested on bovine aged oocytes and no significant differences were found on the obtained cleavage rates. However, a significant increase in the rate of zygotes developed to the blastocyst stage was identified, compared to aged oocytes without L-carnitine [59]. Our results also demonstrate that UA supplementation during physiological maturation improved the cleavage rate in prepubertal and adult females. Although the cleavage rates of UA aged oocytes were not significantly different from control aged oocytes, higher D7 embryo rates were identified in UA aged oocytes from both prepubertal and adult females. These results pointed out that the anti-aging effect of UA previously identified in different cell cultures [28,35] is valid for oocytes and embryos. Oocyte aging is a multifactorial process that impairs the development of the embryo, and restoring the developmental capacity of aged oocytes is an important objective that was attained in the present study.

Additionally, we also assessed the preventing role of UA in the age-related deterioration on embryo quality. Although in this study no significant differences were found on embryo quality rates, the oocytes supplemented with UA increased the number of transferrable embryos, compared to the untreated ones. A previous study reported that L-carnitine treatment could improve the quality of embryos developed from aged bovine oocytes through the reduction in ROS levels and higher levels of glutathione as well as of others antioxidant enzymes [58]. The differences between these results and ours may be due to the different techniques applied to assess embryo quality. The morphological evaluation of embryo quality remains a subjective method that depends on the observer’s experience. In the future, more studies should be addressed using other techniques to accurately assess embryo quality.

It is widely known that oxidative stress plays a crucial role in the age-associated decrease in fertility. Oxidative stress is one of the major contributors to low oocyte maturation efficiency, and oocyte quality deterioration, thereby impairing subsequent embryo development [11]. Intercellular communication between the gamete and somatic cells is crucial for the proper development of high-quality oocytes. Changes in the microenvironment of aged ovaries have been reported, such as decreased antioxidant enzymatic activity leading to an impaired ROS scavenging efficiency. Furthermore, GCs from young and older females were shown to have differentially expressed genes associated to antioxidant activities and maternal age [2,13]. Thus, it is of great importance that found mechanisms manage oxidative stress in order to rescue oocyte from aging and to overcome infertility issues. The Nrf2-Keap1 pathway has been extensively studied due to its capacity to cope with the deleterious effects of oxidative stress and exerting antioxidant proprieties [60]. To further study the activation of the Nrf2 signaling pathway in bovine GCs, we assessed the mRNA expression level of *NFE2L2* and its downstream antioxidant (*NQO1*). Our results showed a significant influence of female age on the level of *NFE2L2* transcripts, which decreases with age. Indeed, greater mRNA expression levels were observed in prepubertal cows when compared to adults. This is in agreement with previous studies that reported a highly expressed level of Nrf2 protein and mRNA on ovarian tissues of childbearing young mice and women from 22 and 49 years old, whereas in aged mice and women a lower expression was found. It was suggested that decreased expression of Nrf2 may be involved in the decline of reproductive capacity of older women and its control may have important implications in delaying ovarian aging [15,61]. Furthermore, Akino and co-workers (2019) showed that the activation of the Nrf2-Keap1 pathway through the administration of dimethylfumarate could reduce ROS levels and lead to delayed infertility [18]. Similar results regarding the effect of UA in reducing ROS in senescent human skin fibroblasts were reported. When these cells were treated with UA, a significant increase in the mRNA expression of *Nrf2* targeted genes, such as *SOD1*, *NQO1*, *GCLC* and *HMOX1* were reported. [35]. In our study, we observed that the expression level of *NFE2L2* and *NQO1* genes in GCs was not significantly affected by UA supplementation. However, further studies should be addressed to confirm the reduction in ROS and subsequent improvement of produced blastocyst, as well as the UA effect on mRNA expression of the aforementioned genes on aged oocytes.

In addition to the damage to mitochondrial DNA (mt-DNA) [25,57], the disruption of mitochondrial gene expression has also been shown to contribute to mitochondrial dysfunction with age. Zhang and colleagues (2019) reported that some genes involved in the OXPHOS, namely mt-ND2, mt-ND3, mt-ND4, mt-ND4L and mt-ND5, were significantly downregulated in the GV stage of oocytes from aged mice, compared with those from young mice [62]. In our study, a no significant difference was found in the *mt-ND5* mRNA expression, between the GCs retrieved from prepubertal and adult cows. Moreover, we also assessed the mt-DNA copy number, because the content of mt-DNA in GCs has been positively associated with oocyte quality and embryo development [63,64]. Although in our study no significant differences were identified between the mt-DNA copy number in GCs from prepubertal and adult cows, or when treated with UA, we observed a greater number of oocytes that developed to the blastocyst stage, compared with the untreated groups. Additionally, Ryu and co-workers (2016) reported that the mt-DNA content and protein level from the respiratory complexes did not change in mice myoblasts supplemented with UA [28]. Further studies should be performed to disclose the mechanism of action of UA in improving female fertility.

## 5. Conclusions

The results obtained in this study confirmed the harmful effect of oocyte aging on its developmental competence. Moreover, our model for aging female gametes proved to be very efficient and useful to study different problems associated with reproductive aging and consequent fertility impairment. Additionally, UA supplementation during the maturation process of aged oocytes improved maturation rates and produced embryos. Therefore, an anti-aging effect of UA in rescuing aged gametes was identified for the first time, improving the blastocyst development, which leads to an increased number of embryos for transfer to recipient females. A positive effect of UA on physiological maturation, MMP and embryonic development was also identified. In conclusion, UA treatment provides a new therapeutic approach to prevent or delay gamete aging, and improve the subsequent blastocyst formation and fertility outcomes in ART.

## Figures and Tables

**Table 1:** Sequences of primer for quantitative reverse-transcription polymerase chain reaction (RT-qPCR), designed specifically for this study.

Gene Symbol	Gene ID	Primers Concentration	Primer Pairs (5′–3′)
*NFE2L2*	497024	200 nM	F: GTCGTCGGGGAGCCTCAAAG R: ATGTCAATCAAATCCATGTCCTGCT
*NQO1*	519632	100 nM	F: CATGGCTGTCAGAAAAGCACTG R: GGTCTGACACAGTGACCTCC
*mt-ND5*	-	75 nM	F: ATTTACAGCAATATGCGCCC R: AAAAGGCGTGGGTACAGATG
*β-actin*	280979	200 nM	F: AGTCGGTTGGATCGAGCATT R: GCTTTTGGGAAGGCAAAGGAC

F, Forward Primer; R, Reverse Primer.

**Table 2:** Effect of Urolithin (UA) supplementation to the maturation medium, cumulus-oocyte-complexes (COCs) aging and female age on the cleavage and day 7 embryo rates (6 sessions, prepubertal: control 22 h, UA 22 h, control aged 30 h, UA aged 30 h; adult: control 22 h, UA 22 h, control aged 30 h, UA aged 30 h groups). D7 embryos, embryos at day 7.

Groups		Oocytes	Cleavage		D7 Embryos	
		(n)	(n)	(%)	(n)	(%)
Prepubertal	Cont 22 h	148	110	74.7 ± 3.6 ^ab^	16	13.8 ± 3.3 ^bc^
	UA 22 h	155	125	80.6 ± 3.2 ^a^	26	20.5 ± 3.6 ^ab^
	Cont Aged 30 h	149	99	66.9 ± 3.9 ^b^	8	7.7 ± 2.6 ^c^
	UA Aged 30 h	144	97	67.2 ± 4.0 ^b^	19	18.9 ± 4.0 ^ab^
Adult	Cont 22 h	155	120	81.4 ± 3.2 ^a^	19	15.9 ± 3.4 ^bc^
	UA 22 h	129	111	80.9 ± 3.4 ^a^	30	26.8 ± 4.3 ^a^
	Cont Aged 30 h	148	112	73.0 ± 3.6 ^ab^	9	7.7 ± 2.6 ^c^
	UA Aged 30 h	138	84	66.5 ± 4.2 ^b^	14	15.9 ± 4.0 ^bc^

Different letters indicate significant differences between groups (*p* ≤ 0.05). Data are expressed as the mean ± standard error mean (SEM).

**Table 3:** Effect of the maturation medium supplementation with Urolithin A (UA), cumulus-oocyte complexes (COCs) aging and female age on the number and quality of the produced embryos. The embryo quality was classified based on morphological criteria as excellent/good (Grade 1), fair (Grade 2) and poor (Grade 3). Prepubertal: control 22 h, UA 22 h, control aged 30 h, UA aged 30 h; adult: control 22 h, UA 22 h, control aged 30 h, UA aged 30 h groups.

Groups		D7 Embryos					
		Grade 1		Grade 2		Grade 3	
		(n)	(%)	(n)	(%)	(n)	(%)
Prepubertal	Control 22 h	5	31.2 ± 11.8	10	58.5 ± 12.9	1	7.3 ± 7.2
	UA 22 h	11	43.4 ± 10.4	13	60.6 ± 10.4	1	1.5 ± 1.7
	Cont Aged 30 h	4	41.5 ± 16.9	4	44.3 ± 17.4	4	0.0 ± 0.0
	UA Aged 30 h	4	20.11 ± 9.2	9	45.9 ± 11.8	6	33.8 ± 12.2
Adult	Control 22 h	8	41.4 ± 11.5	9	45.5 ± 11.9	2	10.0 ± 6.6
	UA 22 h	14	43.8 ± 9.5	12	38.9 ± 9.4	4	13.7 ± 6.7
	Cont Aged 30 h	4	40.7 ± 17.1	4	46.3 ± 17.5	4	10.0 ± 10.0
	UA Aged 30 h	5	33.9 ± 13.0	7	52.0 ± 14.0	2	11.2 ± 8.3

Data are expressed as the mean ± standard error mean (SEM).
